# Smoking-associated DNA methylation markers predict lung cancer incidence

**DOI:** 10.1186/s13148-016-0292-4

**Published:** 2016-11-25

**Authors:** Yan Zhang, Magdeldin Elgizouli, Ben Schöttker, Bernd Holleczek, Alexandra Nieters, Hermann Brenner

**Affiliations:** 1 Division of Clinical Epidemiology and Aging Research, German Cancer Research Center (DKFZ), Heidelberg, Germany; 2 Center for Chronic Immunodeficiency (CCI), Research Group Epidemiology, University Medical Center Freiburg, Freiburg, Germany; 3 Saarland Cancer Registry, Saarbrücken, Germany; 4 Division of Preventive Oncology, German Cancer Research Center (DKFZ) and National Center for Tumor Diseases (NCT), Heidelberg, Germany; 5 German Cancer Consortium (DKTK), German Cancer Research Center (DKFZ), Heidelberg, Germany

**Keywords:** DNA methylation, Lung cancer, Smoking, Risk prediction, *AHRR*, *F2RL3*

## Abstract

**Background:**

Newly established blood DNA methylation markers that are strongly associated with smoking might open new avenues for lung cancer (LC) screening. We aimed to assess the performance of the top hits from previous epigenome-wide association studies in prediction of LC incidence.

In a prospective nested case-control study, DNA methylation at *AHRR* (cg05575921), *6p21.33* (cg06126421), and *F2RL3* (cg03636183) were measured by pyrosequencing in baseline whole blood samples of 143 incident LC cases identified during 11 years of follow-up and 457 age- and sex-matched controls without diagnosis of LC until the end of follow-up. The individual and joint associations of the 3 markers with LC risk were estimated by logistic regression, adjusted for potential confounders including smoking status and cigarette pack-years. The predictive performance was evaluated for both the individual markers and their combinations derived from multiple algorithms.

**Results:**

Pronounced demethylation of all 3 markers was observed at baseline among cases compared to controls. Risk of developing LC increased with decreasing DNA methylation levels, with adjusted ORs (95% CI) of 15.86 (4.18–60.17), 8.12 (2.69–4.48), and 10.55 (3.44–32.31), respectively, for participants in the lowest quartile of *AHRR*, *6p21.33*, and *F2RL3* compared to participants in the highest 2 quartiles of each site among controls. The individual 3 markers exhibited similar accuracy in predicting LC incidence, with AUCs ranging from 0.79 to 0.81. Combination of the 3 markers did not improve the predictive performance (AUC = 0.80). The individual markers or their combination outperformed self-reported smoking exposure particularly in light smokers. No variation in risk prediction was identified with respect to age, follow-up time, and histological subtypes.

**Conclusions:**

*AHRR*, *6p21.33*, and *F2RL3* methylation in blood DNA are predictive for LC development, which might be useful for identification of risk groups for further specific screening, such as CT examination.

**Electronic supplementary material:**

The online version of this article (doi:10.1186/s13148-016-0292-4) contains supplementary material, which is available to authorized users.

## Background

Lung cancer (LC) accounts for approximately 20% of all cancer deaths worldwide [1]. The mortality rate is close to the incidence rate (ratio of mortality to incidence is 0.87) [1, 2], reflecting the poor prognosis that results from the predominant diagnosis of late-stage disease. It thus has been a long-standing goal to establish an effective non-invasive screening tool for LC. DNA methylation markers detected in body fluids have rapidly emerged as promising candidates [3–5]. Many studies have demonstrated the diagnostic efficiency of DNA hypermethylation of a variety of well-known cancer-related genes, such as *p16*, *RASSF1*, *APC*, *MGMT*, *DAPK*, *GATA5*, and *HOX9*, in various biofluids, including bronchial aspirates, sputum, serum, plasma, and cell-free circulating DNA [3, 4, 6]. A commercial product, Epi proLung *SHOX2* methylation assay, has already become available [7].

Recently, epigenome-wide association studies (EWAS) have opened a new avenue for LC screening, in that hundreds of highly reproducible blood DNA methylation markers were linked to smoking [8], the major risk factor of LC. The top signal from previous EWAS was cg05575921 in the aryl-hydrocarbon receptor repressor (*AHRR*) gene [8], known as a tumor repressor and key regulator for metabolizing carcinogens from tobacco smoke, such as dioxin toxicity [9, 10]. *AHRR* (cg05575921) was also found to be hypomethylated and overexpressed in the lung tissue of smokers [11]. Cg03636183 in coagulation factor II (thrombin) receptor-like 3 (*F2RL3*) was the first EWAS-discovered locus [12], which was likewise consistently confirmed by multiple EWAS since then [8]. The *F2RL3* gene (also known as PAR-4) codes a protein involved in inflammatory reactions and blood coagulation [13]. Hypercoagulation is a common process observed in tumorigenesis, including LC [14]. Cg06126421 located at intergenic region of *6p21.33* was another top-ranked locus associated with smoking [8]. Our previous investigations focused on these top-ranked loci have demonstrated that *F2RL3* methylation is a strong predictor for both LC incidence and mortality [15], and smoking-induced hypomethylation at cg05575921 in *AHRR* and cg06126421 in *6p21.33* are strongly associated with increased risk of overall cancer death [16]. To further corroborate and expand evidence of smoking-associated DNA methylation in prediction of LC risk, we assessed the individual and joint associations of blood DNA methylation at *AHRR*, *6p21.33*, and *F2RL3* with LC incidence in a case-control study nested in the Epidemiologische Studie zu Chancen der Verhütung, Früherkennung und optimierten Therapie chronischer Erkrankungen in der älteren Bevölkerung (ESTHER) cohort.

## Methods

### Study population and data collection

ESTHER, a population-based cohort study, was established to investigate new avenues of prevention, early detection, and optimal treatment of chronic diseases in the elderly [17]. The cohort consists of 9949 participants (50–75 years of age at baseline), recruited by their general practitioners during routine health checkups between July 2000 and December 2002 in Saarland, Germany, and followed up thereafter. At baseline, participants completed a standardized self-administered questionnaire (collecting information on sociodemographic characteristics, lifestyle factors, and history of major diseases) and donated biological samples (blood, stool, urine). In addition, comprehensive medical data, such as medical diagnoses and drug prescriptions, were obtained from the general practitioners’ reports. All participants provided written informed consent. The study was approved by the ethics committees of the University of Heidelberg and of the state medical board of Saarland, Germany.

For the current analysis, a nested case-cohort study was conducted within the ESTHER cohort. A total of 150 incident LC cases (International Classification of Diseases-10 (ICD-10)-code C34) were identified during follow-up between 2000 and end of 2012 through record linkage with the Saarland Cancer Registry, which registers ≥95% of all LC cases in the underlying population. Three controls, matched to each case by age and sex, were selected from ESTHER participants without diagnosis of LC until the end of 2012. Seven cases without sufficient DNA available for laboratory measurements were excluded. The time interval between blood sample collection at enrollment and diagnosis of LC ranged from 1 month to 11 years [median (interquartile range), 5.2 years (2.9–7.9)].

### Methylation assessment

Whole blood DNA methylation at *AHRR* [cg05575921 (Chr5: 373378; GRCh37/hg19)], 6p21.33 [cg06126421 (Chr6: 30720081; GRCh37/hg19)], and *F2RL3* [cg03636183 (chr19: 17000586; GRCh37/hg19)] was quantified by pyrosequencing on the PyroMark Q96 MD apparatus (Qiagen GmbH, Hilden, Germany). Samples were randomized in 96-well plates (with cases and controls equally represented in each plate) and analyzed in a blinded fashion in the same laboratory. Each assay included non-cytosine-phosphate-guanine (CpG) cytosines as internal controls to verify efficient bisulfite conversion. The primers for the pyrosequencing analyses are provided in Additional file 1: Table S1. The quantitative performance of the pyrosequencing assays was assessed by measuring DNA methylation standards of known proportions of unmethylated (whole genome amplified) and fully methylated (Universal Methylated Human DNA Standards, Zymo Research Europe GmbH, Freiburg, Germany) genomic DNA and optimized by means of an annealing temperature gradient. DNA methylation standards were included in each plate run. PCR products were rendered single stranded according to an established protocol. Three picomoles of sequencing primer was used to perform the pyrosequencing reaction on the PyroMark Q96 MD apparatus (Qiagen GmbH, Hilden, Germany). The percentage methylation at each CpG was calculated using the PyroMark CpG Software v.1.0.11 build 14 (Qiagen GmbH, Hilden, Germany).

### Statistical analysis

Participants were assigned into training and validation sets according to time points of laboratory measurement. The training set consisted of 78 cases and 222 controls who were enrolled during initial 9 months of recruitment (July 2000–March 2001) and had DNA samples firstly available and measured first. The validation set consisted of 65 cases and 235 controls who were enrolled in the later period of recruitment (April 2001–December 2002) and had DNA methylation measurements approximately 6 months later. The characteristics of the study populations by case-control status are described separately for the training and validation sets. Differences between cases and controls were assessed by chi-square test for categorical variables and by Wilcoxon-Mann-Whitney test for continuous variables.

The associations of individual methylation markers (*AHRR*_cg05575921, *6p21.33*_cg06126421, *F2RL3*_cg03636183) with LC incidence were estimated by unconditional logistic regression in both training and validation samples, with adjustment for age and sex only in model 1; additionally for smoking status (never smoker, former smoker, current smoker) and lifetime cumulative smoking intensity (pack-years) in model 2; and further for the following potential confounders in model 3 (fully adjusted model): body mass index (BMI, kg/m^2^), physical activity [inactive, low, medium/high (defined as follows: inactive, <1 h/week of physical activity; medium/high, ≥2 h/week of vigorous physical activity or ≥2 h/week of light physical activity; low, other)], systolic blood pressure (mmHg), total cholesterol level (mg/dL), and prevalence of hypertension (yes/no), cardiovascular disease (yes/no), diabetes (yes/no), and cancer (yes/no) at baseline. DNA methylation at the 3 CpGs were entered into the models either as continuous variables (calculating odds ratios for a decrease in methylation by 1 standard deviation) or as categorical variables (participants classified according to quartiles of each CpG site among controls in the training set and using the 3rd and 4th quartile altogether as the reference). Dose-response relationships between methylation at the 3 CpGs and LC incidence were assessed by restricted cubic spline (RSC) regression [18], again controlling for the above listed confounders. Potential interactions between DNA methylation at the target sites and those covariates were evaluated by including pertinent product terms in the fully adjusted models. No statistically significant interactions were detected. The associations of the individual methylation markers with incident LC were furthermore examined separately among heavy smokers (participants with ≥30 pack-years of smoking who were either current smokers or had quit smoking ≤15 years ago) and light smokers (participants with <30 pack-years of smoking or former smokers who had quit smoking >15 years ago).

**Fig. 1:**
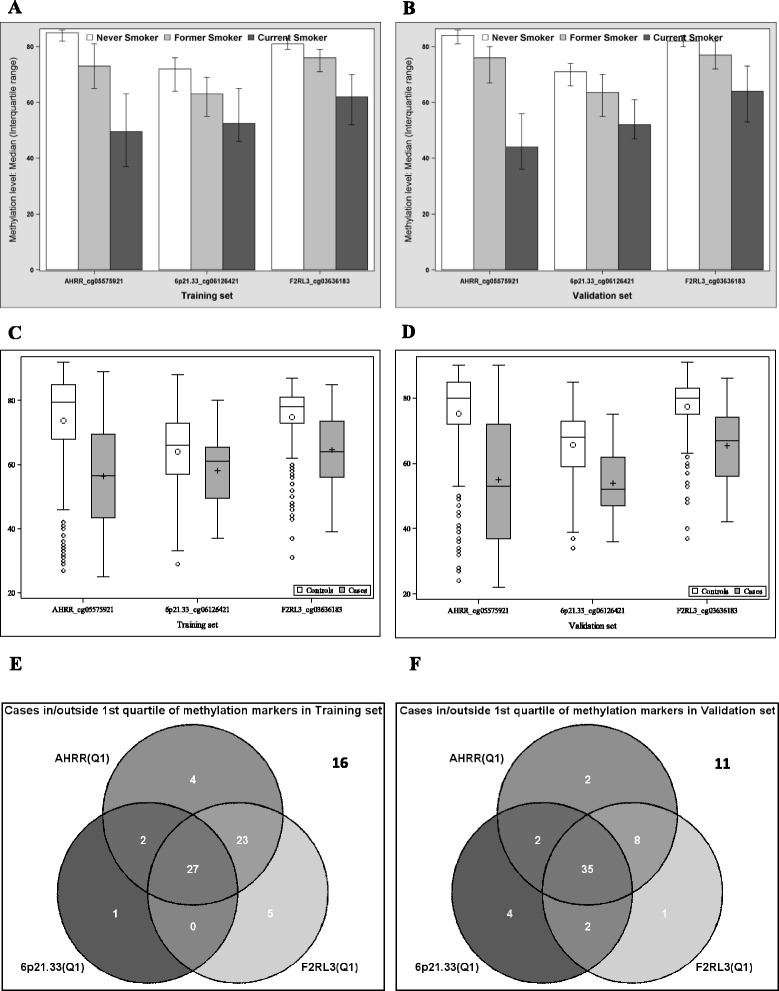
Methylation distribution at baseline by smoking status and lung cancer status. **a**, **b** Present methylation levels of *AHRR*_cg05575921, *6p21.33*_cg06126421, and *F2RL3*_cg03636183 among current, former, and never smokers at baseline, respectively, in the training and validation set. **c**, **d** Present methylation levels of *AHRR*_cg05575921, *6p21.33*_cg06126421, and *F2RL3*_cg03636183 among lung cancer cases and controls, respectively, in the training set and validation set. **e**, **f** Illustrate distribution of lung cancer cases inside and outside the first quartile of methylation among controls at *AHRR*_cg05575921, *6p21.33*_cg06126421, and *F2RL3*_cg03636183, respectively, in the training and validation set

The performance of the 3 individual methylation markers in predicting incident LC was examined by areas under the curve (AUC) in the training set and then tested in the validation set through applying regression coefficients derived from analyses in the training set. Multiple algorithms for combining the 3 markers were employed as follows: (a) additive and non-additive combinations of the markers were included in a logistic regression model containing the following terms: *β*1 × *M*_*AHRR*_ + *β*2 × *M*_*6p21.33*_ + *β*3 × *M*_*F2RL3*_ + *β*4 × Interaction1 + β5 × Interaction2 + β6 × Interaction3, where *β* refers to the logistic regression coefficient of each CpG, *M* refers to the methylation level of the corresponding site, and Interaction refers to non-linear interactions between each pair of sites; (b) methylation of the 3 markers was integrated into a smoking index according to an algorithm introduced by Teschendorff et al. [19]; (c) given that ≥80% cases occurred in the lowest quartiles of 3 CpGs (Venn diagram in Fig. 1), a methylation score based on 3 markers was built, with values of 3, 2, 1, and 0, respectively, for participants in the lowest quartiles of all 3 CpGs, of 2 of the 3 CpGs, of 1 of the 3 CpGs, and others; (d) optimal cut points of each CpG were determined by Youden’s J Index [20], and 3 binary methylation variables were simultaneously fitted in a regression model. Again, all combination algorithms were first derived in the training set and subsequently tested in the validation set. All analyses were repeated and stratified by smoking history (heavy and light smokers as defined above), by 2 major age groups (<65 and ≥65 years), by time distance from blood sample collection to diagnosis (initial 5 years after recruitment and later years), and by histological subtypes of LC [small cell lung cancer (SCLC) and non-small cell lung cancer (NSCLC; adenocarcinoma/squamous cell carcinoma/others)]. Stratified analyses were conducted in the whole dataset (training and validation set combined), with correction for potential overoptimism by leave-one-out cross-validation.

All statistical analyses were conducted using SAS 9.3 (SAS Institute, Cary, NC), and 2-sided *p* values of <0.05 were considered statistically significant.

## Results

The distributions of sociodemographic characteristics, lifestyle factors, and history of major chronic diseases among LC cases and controls at baseline enrollment are presented in Table 1. In the training set, mean age was 64 years for both cases and controls. There were many more males (>70%) and then females (<30%) among both cases and controls. Current smokers, low education, and physical inactivity were more common among cases than among controls. No statistically significant differences were seen for BMI, family history of cancer, and prevalence of chronic diseases. Similar distributions of the characteristics among cases and controls were also observed in the validation sample. The time interval between blood sample collection and diagnosis ranged from 2 months to 11 years [median (interquartile range), 5.5 (3.2–8.1)] for 78 cases in the training set and from 1 month to 10 years [median (interquartile range), 4.9 (2.6–7.3)] for 65 cases in the validation set.

**Table 1:** Characteristics of the study population

Characteristics	Training set			Validation set		
	Cases (*N* = 78)	Controls (*N* = 222)	*p* value^b^	Cases (*N* = 65)	Controls (*N* = 235)	*p* value^b^
	No. (%)^a^	No. (%)^a^		No. (%)^a^	No. (%)^a^	
Age (years)	64 (5.7)	64 (6.1)		64 (5.9)	64 (6.3)	
Sex						
Male	58 (74.4)	167 (75.2)		48 (73.9)	169 (71.9)	
Female	20 (25.6)	55 (24.8)	0.88	17 (26.1)	66 (28.1)	0.76
Smoking status^c^						
Never smoker	5 (6.5)	86 (39.8)		9 (13.9)	100 (44.8)	
Former smoker	29 (37.7)	90 (41.7)		26 (40.0)	88 (39.5)	
Current smoker	43 (55.8)	40 (18.5)	<0.0001	30 (46.2)	35 (15.7)	<0.0001
Body mass index (kg/m^2^)^d^						
Under weight (<18.5)	1 (1.3)	0		1 (1.6)	1 (0.43)	
Normal weight (18.5–<25.0)	25 (32.5)	55 (24.8)		19 (29.2)	62 (26.4)	
Overweight (25.0–<30.0)	29 (37.7)	115 (51.8)		32 (49.2)	119 (50.6)	
Obesity (≥30.0)	22 (28.5)	52 (23.4)	0.07	13 (20.0)	53 (22.6)	0.74
Educational level^e^						
Low	59 (78.7)	143 (65.3)		57 (87.7)	164 (71.6)	
Intermediate	11 (14.7)	41 (18.7)		3 (4.6)	35 (15.3)	
High	5 (6.6)	35 (16.0)	0.06	5 (7.7)	30 (13.1)	0.02
Physical activity^f^						
Inactive	18 (23.1)	40 (18.0)		25 (38.5)	48 (20.6)	
Insufficient	43 (55.1)	95 (42.8)		23 (35.4)	115 (49.4)	
Sufficient	17 (21.8)	87 (39.2)	0.02	17 (26.1)	70 (30.0)	0.01
Family history of cancer^g^						
No	39 (52.0)	132 (60.0)		30 (47.6)	132 (56.4)	
Yes	36 (48.0)	88 (40.0)	0.23	33 (52.4)	102 (43.6)	0.21
Diabetes^h^						
Not prevalent	64 (82.0)	188 (85.1)		50 (76.9)	198 (84.3)	
Prevalent	14 (18.0)	33 (14.9)	0.53	15 (23.1)	37 (15.7)	0.17
Cardiovascular disease						
Not prevalent	60 (76.9)	177 (79.7)		44 (67.7)	180 (76.6)	
Prevalent	18 (23.1)	45 (20.3)	0.60	21 (32.3)	55 (23.4)	0.14
Systolic blood pressure (mmHg)^i^	140 (18)	140 (19)	0.12	141 (17)	141 (19)	0.77
Total cholesterol (mg/dL^)j^	205.6 (54.4)	200.5 (58.7)	0.48	236.1 (38.4)	224.8 (43.6)	0.03
Pack-years^k^	39.2 (25.4)	16.2 (20.2)	<0.0001	34.3 (22.6)	13.4 (18.4)	<0.0001

^a^Table shows numbers (proportions) for categorical variables and means (standard deviation) for continuous variables

^b^Chi-square test for categorical variable and Wilcoxon test for continuous variables

^c^Data missing for 1 case and 6 controls in the training set and 12 controls in the validation set

^d^Data missing for 1 case in the training set

^e^Data missing for 3 cases and 3 controls in the training set and 6 controls in the validation set

^f^Data missing for 2 controls in the training set

^g^Data missing for 2 cases and 3 controls in the training set and 2 cases and 1 control in the validation set

^h^Data missing for 1 control in the training set

^i^Data missing for 4 cases and 5 controls in the training set and 2 cases and 4 controls in the validation set

^j^Data missing for 1 controls in the training set and 2 controls in the validation set

^k^Data missing for 2 cases and 27 controls in the training set and 3 cases and 27 controls in the validation set

DNA methylation levels at *AHRR*_cg05575921, *6p21.33*_cg06126421, and *F2RL3*_cg03636183 were mutually correlated (Additional file 1: Figure S1), and consistent patterns were observed in both the training and the validation set (Spearman correlation coefficients, 0.62–0.79). Figure 1 depicts methylation levels of the 3 markers among current, former, and never smokers as well as among LC cases and controls. For all 3 markers, current smokers showed lower methylation levels than never smokers, and former smokers had intermediate methylation levels (Fig. 1a, b). In addition, at baseline, cases exhibited strikingly lower methylation levels than controls (Fig. 1c, d). Venn diagrams in Fig. 1d, e, respectively, illustrate that 62 of 78 cases in the training set and 54 of 65 cases in the validation set had methylation levels in the lowest quartiles of any of the 3 markers among controls.

**Fig. 2:**
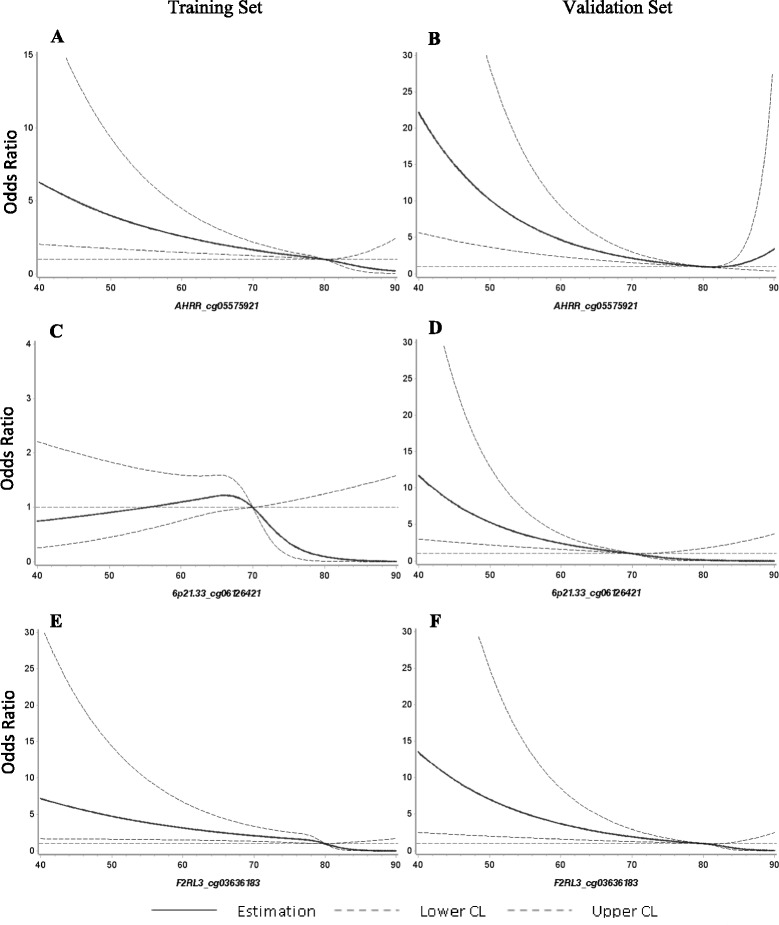
Dose-response *curves* of methylation at *AHRR*, *6p21.33*, and *F2RL3* with lung cancer incidence. **a**.**b**. present the dose-response curves for AHRR_cg05575921, respectively, in training and validation set. **c**.**d**. present the dose-response curves for 6p21.33_cg06126421, respectively, in training and validation set. **e**.**f**. present the dose-response curves for F2RL3_cg03636183, respectively, in training and validation set

Table 2 shows the individual associations of the 3 methylation markers with LC incidence in the validation set. Age- and sex-adjusted odds ratios (ORs) (95% confidence interval (CI)) for participants with methylation levels in the lowest quartiles of *AHRR*_cg05575921, *6p21.33*_cg06126421, and *F2RL3*_cg03636183 were 23.93 (9.61–59.57), 15.55 (6.89–35.10), and 19.25 (8.59–43.15), respectively, compared to those in the higher 2 quartiles of each site of controls. Adjustment for smoking status and pack-years reduced the corresponding OR estimates to 17.17 (4.91–60.03), 6.92 (2.63–18.18), and 10.84 (4.03–29.19). Further controlling for a variety of potential confounding factors did not substantially alter the associations, with 16-, 8-, and 11-fold risk of developing LC observed correspondingly. In addition, a decrease in methylation by 1 standard deviation of each site was associated with approximately doubled LC risk. Dose-response analyses disclosed a monotonous decrease of LC incidence with increasing methylation at all 3 CpGs (Fig. 2). Similar results were also derived in the training samples for analyses of *AHRR* and *F2RL3* methylation (Additional file 1: Table S2). Table 3 shows the associations of current and past smoking with incident LC, which were attenuated from an OR of 3.07 (0.93–10.15) for current smokers and 1.58 (0.54–4.60) for former smokers to null results when controlling for any of the 3 methylation markers. These patterns suggest that the association between smoking exposure and LC development might be partly mediated by methylation at those 3 CpGs. Smoking-status stratified analyses yielded stronger associations of the 3 CpGs with LC incidence in light smokers than in heavy smokers (Additional file 1: Table S3).

**Table 2:** Associations of methylation at *AHRR*, *6p21.33*, and *F2RL3* with lung cancer incidence in the validation set

CpG site	Methylation level^a^	Controls	Cases	OR (95% CI)		
				Model 1^b^	Model 2^c^	Model 3^d^
*AHRR*_cg05575921	≥85 (quartile 4)	59	6	Ref.	Ref.	Ref.
	<85 (quartile 3)	73	1			
	<80 (quartile 2)	58	11	4.13 (1.48–11.52)	3.70 (1.12–12.22)	4.63 (1.27–16.80)
	<68 (quartile 1)	45	47	23.93 (9.61–59.57)	17.17 (4.91–60.03)	15.86 (4.18–60.17)
	Per SD less methylation		–	2.61 (2.02–3.37)	2.58 (1.69–3.94)	2.37 (1.46–3.85)
*6p21.33*_cg06126421	≥73 (quartile 4)	63	4	Ref.	Ref.	Ref.
	<73 (quartile 3)	76	6			
	<66 (quartile 2)	50	12	3.90 (1.52–9.98)	3.00 (1.06–8.48)	4.08 (1.27–13.07)
	<57 (quartile 1)	46	43	15.55 (6.89–35.10)	6.92 (2.63–18.18)	8.12 (2.69–24.48)
	Per SD less methylation		–	2.92 (2.15–3.98)	2.11 (1.45–3.05)	2.11 (1.39–3.19)
*F2RL3*_cg03636183	≥81 (quartile 4)	113	5	Ref.	Ref.	Ref.
	<81 (quartile 3)	39	5			
	<78 (quartile 2)	40	9	3.91 (1.45–10.55)	2.75 (0.91–8.37)	2.45 (0.72–8.31)
	<73 (quartile 1)	43	46	19.25 (8.59–43.15)	10.84 (4.03–29.19)	10.55 (3.44–32.31)
	Per SD less methylation		–	2.46 (1.90–3.19)	1.86 (1.33–2.60)	1.72 (1.17–2.51)

*Abbreviations*: *OR* odds ratio, *CI* confidence interval, *Ref*. reference category, *SD* standard deviation

^a^Quartiles of each site among controls in the training set

^b^Model 1: adjusted for age and sex

^c^Model 2: like model 1, additionally adjusted for smoking status and pack-years

^d^Model 3: like model 2, additionally adjusted for educational level, BMI, physical activity, systolic blood pressure, total cholesterol, family history of cancer, prevalence of hypertension, cardiovascular disease, and diabetes

**Table 3:** Associations of smoking with lung cancer incidence in the validation set

Smoking exposure	Controls	Cases	OR (95% CI)			
			Model 1^a^	Model 2^b^	Model 3^c^	Model 4^d^
Never smoker	100	9	Ref.	Ref.	Ref.	Ref.
Former smoker	88	26	1.58 (0.54–4.60)	0.94 (0.27–3.21)	1.05 (0.33–3.30)	1.08 (0.33–3.51)
Current smoker	35	30	3.07 (0.93–10.15)	0.81 (0.21–3.15)	1.35 (0.36–5.06)	1.07 (0.28–4.09)
Per 21 (=1SD) pack-years		–	2.26 (1.46–3.51)	1.55 (0.96–2.48)	1.93 (1.21–3.07)	1.72 (1.08–2.75)

*Abbreviations*: *OR* odds ratio, *CI* confidence interval, *Ref*. reference category, *SD* standard deviation

^a^Model 1: adjusted for age and sex

^b^Model 2: adjusted for age, sex, and methylation of *AHRR*_cg05575921

^c^Model 3: adjusted for age, sex, and methylation of *6p21.33*_cg06126421

^d^Model 4: adjusted for age, sex, and methylation of *F2RL3*_cg03636183

**Fig. 3:**
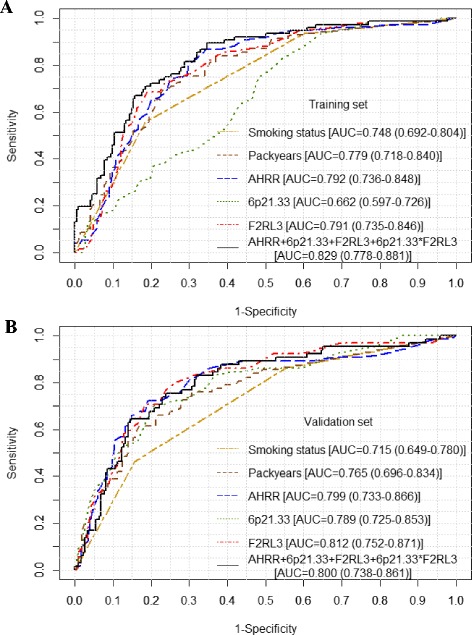
Receiver operating characteristic (ROC) *curves* for methylation at *AHRR*, *6p21.33*, and *F2RL3* in discrimination of incident lung cancer in training set (panel **a**) and in validation set (panel **b**). ROC *curves* for self-reported smoking status and pack-years are shown for comparison

**Fig. 4:**
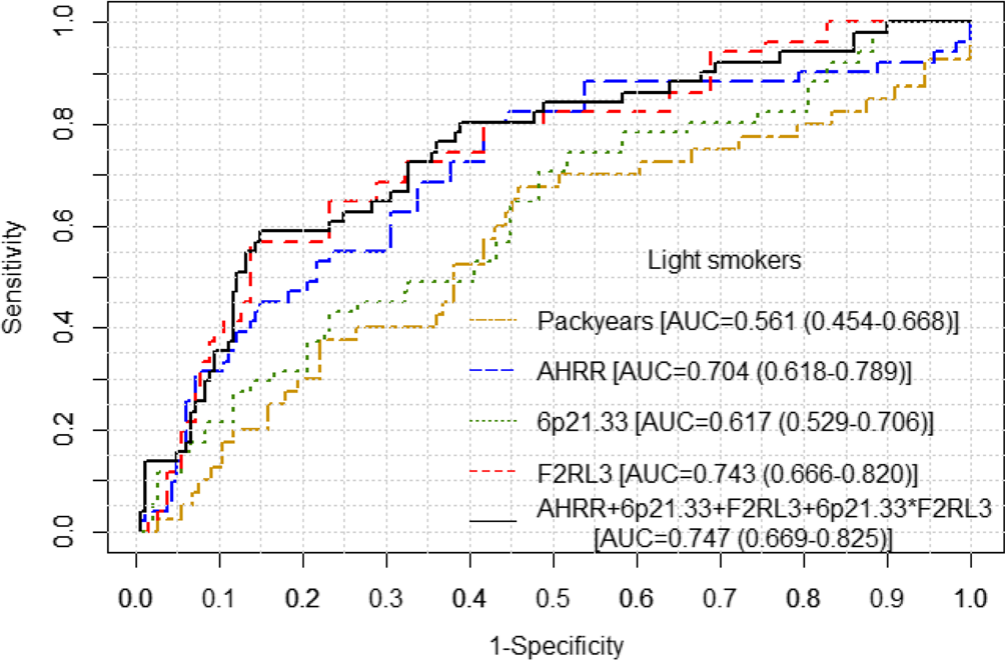
Receiver operating characteristic (ROC) curves for methylation at *AHRR*, *6p21.33*, and *F2RL3* and pack-years in discrimination of incident lung cancer among light smokers

The predictive performance of the 3 methylation markers and their combinations are presented in Table 3 and Additional file 1: Table S4. Applying regression coefficients derived from the training set, the AUCs of the 3 individual markers in the validation set were similar, ranging from 0.789 to 0.812, and larger than AUCs of self-reported smoking exposure [smoking status (AUC = 0.715) or pack-years (AUC = 0.764) in the validation set]. When combining the 3 markers, statistically significant interaction was detected between *6p21.33*_cg06126421 and *F2RL3*_cg03636183 (*p* < 0.0001). The training set yielded a combination algorithm as (−0.0685) × cg05575921 + 0.4673 × cg06126421 + 0.3173 × cg03636183 + (−0.00612) × cg06126421 × cg03636183. Application of this algorithm in the validation set resulted in an AUC (95% CI) of 0.800 (0.737–0.861). Corresponding receiver operating characteristic (ROC) curves derived from methylation markers as well as from self-reported smoking exposure are presented in Fig. 3. Combining the 3 markers by the other methylation algorithms outlined in the methods section yielded very similar predictive performance (AUCs, 0.788–0.819; Additional file 1: Table S4). In smoking-status stratified analyses, neither self-reported smoking exposure (lifetime pack-years) nor methylation markers were able to predict occurrence of LC among heavy smokers (overoptimism corrected AUCs, 0.504–0.587; Additional file 1: Table S5). However, among light smokers, the methylation markers (*AHRR*_cg05575921, *F2RL3*_cg03636183, and the 3 marker combinations) showed substantially superior performance compared to pack-years (AUCs, 0.704–0.747 vs. 0.561, *p* values <0.05; Additional file 1: Table S5 and Fig. 4). Consistent performance of either individual or combined markers was also observed in age-specific and follow-up time-specific analyses (Table 4). The AUCs for NSCLC (AUC = 0.823), in particular for adenocarcinoma (AUC = 0.830), were tentatively larger compared to SCLC (AUC = 0.739). However, these differences did not reach statistical significance (*p* > 0.05).

**Table 4:** Individual and joint discriminative performance of methylation at *AHRR*, *6p21.33*, and *F2RL3*

Group	AUC (95% CI)			
	*AHRR*_cg05575921	*6p21.33*_cg06126421	*F2RL3*_cg03636183	Combination^a^
Overall				
Training set (*n* = 78 cases)	0.792 (0.736–0.848)	0.662 (0.597–0.726)	0.791 (0.735–0.846)	0.829 (0.778–0.881)
Validation set (*n* = 65 cases)	0.799 (0.733–0.866)	0.789 (0.725–0.853)	0.812 (0.725–0.871)	0.800 (0.737–0.861)
Age specific prediction				
<65 years (*n* = 77 cases)	0.789 (0.728–0.850)	0.745 (0.687–0.803)	0.792 (0.735–0.849)	0.800 (0.745–0.856)
≥65 years (*n* = 66 cases)	0.790 (0.726–0.856)	0.677 (0.604–0.751)	0.793 (0.732–0.854)	0.817 (0.760–0.875)
Follow-up time-specific prediction				
Initial 5 years (*n* = 68 cases)	0.791 (0.733–0.849)	0.696 (0.631–0.761)	0.808 (0.758–0.857)	0.812 (0.759–0.865)
Later years (*n* = 75 cases)	0.791 (0.734–0.849)	0.730 (0.673–0.786)	0.779 (0.722–0.837)	0.807 (0.755–0.859)
Histological subtype prediction				
SCLC (*n* = 22 cases)	0.744 (0.630–0.858)	0.651 (0.535–0.767)	0.738 (0.632–0.843)	0.739 (0.634–0.844)
NSCLC (*n* = 119 cases)	0.802 (0.758–0.847)	0.721 (0.672–0.770)	0.798 (0.754–0.843)	0.823 (0.782–0.864)
Adenocarcinoma (*n* = 48 cases)	0.814 (0.751–0.877)	0.730 (0.659–0.800)	0.814 (0.751–0.876)	0.830 (0.770–0.891)
Squamous cell carcinoma (*n* = 38 cases)	0.787 (0.709–0.864)	0.731 (0.655–0.807)	0.769 (0.699–0.839)	0.786 (0.717–0.856)
Others (*n* = 32 cases)	0.775 (0.686–0.864)	0.673 (0.576–0.770)	0.800 (0.713–0.888)	0.813 (0.729–0.896)

*Abbreviations*: *AUC* areas under the curve, *CI* confidence interval, *SCLC* small cell lung cancer, *NSCLC* non-small cell lung cancer

^a^Combination formula: *β*1 × *M*_*AHRR*_ + *β*2 × *M*_*6p21.33*_ + *β*3 × *M*_*F2RL3*_ + *β*4 × *M*_*6p21.33*_ × *β*3 × *M*_*F2RL3*_ = (−0.0685) × cg05575921 + 0.4673 × cg06126421 + 0.3173 × cg03636183 + (−0.00612) × cg06126421 × cg03636183, where underlined coefficients were derived from regression coefficients in training set

## Discussion

In this nested case-control study, we demonstrated prospective associations of hypomethylation at *AHRR*, *6p21.33*, and *F2RL3* with LC incidence, which persisted after controlling for lifetime cumulative smoking exposure and various other potential confounders, whereas the strong association of current smoking with incident LC disappeared after adjustment for any of the 3 methylation markers. Each of the 3 individual markers as well as their combination was highly predictive of LC risk, with an AUC of approximately 0.80. Similarly high predictive accuracies of either individual or combined markers were also observed in specific subgroups defined by age, follow-up time, and histological subtypes.

The 3 target loci of the current study were the top signals related to tobacco smoking in previous EWAS conducted in various independent populations [19, 21–28]. Demethylation at both *AHRR*_cg05575921 (≤77%) and *6p21.33*_cg06126421 (≤60%) was found to be associated with a 2.5-fold risk of dying from any cancer in our previous study [16]. In addition, methylation of a CpG site within *F2RL3*, adjacent to *F2RL3*_cg03636183, alone predicted LC incidence with an AUC of 0.77 in our previous cohort study of 5000 ESTHER participants [15]. These findings are corroborated and expanded by our current findings, which were derived from a larger number of LC cases with DNA methylation being assessed by a different method that is regarded as a gold-standard technique for methylation analyses at specific sites [29]. During preparation of the current manuscript, an EWAS conducted in pre-diagnostic blood samples of LC cases and controls was published, where *AHRR*_cg05575921, *6p21.33*_cg06126421, and *F2RL3*_cg03636183 methylation were again ranked as the top CpGs inversely associated with LC risk [30]. The researchers further validated these associations in 664 case-control pairs matched for smoking from another 3 large cohorts. Consistent with our findings, they also reported that AUC increased to 0.78 when adding *AHRR*_cg05575921 and *F2RL3*_cg03636183 to the model with smoking status alone (AUC = 0.71). Taken together, there is rapidly accumulating evidence indicating that DNA methylation levels of the 3 target sites are highly reliable and informative markers for future development of LC.

Previous studies evaluating the performance of DNA methylation of cancer-related genes have suggested that methylation panels with multiple genes provide improved sensitivity and specificity for discriminating LC cases from controls [31–33]. In the current study, although we explored multiple algorithms to combine the 3 methylation markers, no gain was obtained in predictive performance. This is probably because all the 3 markers are closely related to smoking exposure and highly correlated with each other. Nevertheless, we identified an unexpected interaction between 2 of the 3 markers. While this interaction is hard to explain by known biological pathways, it deserves further exploration and confirmation in future studies. On the other hand, the current study confirmed via training and validation that all 3 markers are equally predictive for LC.

A few other DNA methylation markers emerged as promising candidates for improving LC diagnosis efficiency in previous studies. For example, for *SHOX2* methylation, a marker which has received CE in vitro diagnostic (IVD) certification, 60% sensitivity and 90% specificity were reported in a study conducted in plasma samples [34]. Even higher sensitivity and specificity of *SHOX2* methylation were reported in studies assessing bronchial aspirates by Schmidt et al. (68% sensitivity and 95% specificity) [35] and by Dietrich et al. (78% sensitivity and 96% specificity) [7]. A panel incorporating methylation of *p16*, *TERT*, *WT1*, and *RASSF1* exhibited 82% sensitivity and 91% specificity in bronchial washings [31]. Performance of these markers appears superior to the performance of the smoking-associated DNA methylation markers assessed in our study. However, these studies evaluated the markers’ performance in retrospective studies with cases already diagnosed as LC and biospecimen collected at/after diagnosis, while the 3 smoking-associated markers were evaluated in prospectively collected samples either in the current study or in the EWAS by Fasanelli et al. [30]. The average time interval between sample collection and diagnosis of LC was 5.3 years in the current study and 3.8–9.6 years in the 4 case sets of Fasanelli’s study [30]. Notably, these 3 smoking-associated markers even outperformed a methylation panel of 6 cancer-related genes (*p16*, *MGMT*, *DAPK*, *RASSF1A*, *PAX5- β*, and *GATA5*) assessed in sputum samples collected 3 to 18 months prior to LC diagnosis (sensitivity and specificity of 64%) [36].

Low-dose computed tomography (CT) screening has been shown to be effective in reducing LC mortality in the National Lung Screening Trial (NLST) [37]. Guided by the NLST and subsequent validation [38], a recommendation has been made by the United States Prevention Service Task Force (USPSTF) to screen high-risk smokers and ex-smokers (55 to 80 years of age, with ≥30 pack-years of smoking and who quit ≤15 years ago if ex-smokers) [39]. Following these criteria, we stratified ever smokers as heavy smokers and light smokers in our study and observed that approximately 40% of LC cases among smokers occurred in light smokers. Of note, substantial predictive performance among light smokers was observed for methylation markers but not for pack-years, suggesting that these methylation markers might be useful for identifying high-risk light smokers for further specific screening. A potential explanation could be that these markers more accurately reflect the overall biologically effective dose of smoking exposure accumulated during lifetime, whereas smoking exposure measurements based on self-reports, including pack-years, may be subject to inaccuracies, e.g., due to recall bias, intentional under-reporting, or discrepancy between inhaled smoke and actually absorbed smoke. The lack of predictive value of the methylation markers among heavy smokers is consistent with and might be explained by our previous findings that methylation alteration at those sites plateaued or saturated among individuals with >30 pack-years of smoking exposure [16, 40].

In addition, DNA methylation is tissue specific, which may have contributed to the observed difference between smoking-associated methylation markers that were assessed in whole blood DNA in our/other study [30] and markers exhibiting superior performance mainly in bronchial washings. Recently, Teschendorff et al. compared smoking-induced methylation changes in buccal and blood samples and demonstrated that the smoking signature defined by methylation candidates from buccal cells outperformed the signature defined by candidates from blood cells in discrimination of 14 of 15 types of epithelial cancer, including LC, and head and neck cancer [19]. This study indicates that biospecimen with direct exposure to smoking, such as buccal, or saliva samples or bronchial aspirates might be more appropriate tissue for identification of candidate markers. Thus, the performance of *AHRR*_cg05575921, *6p21.33*_cg06126421, and *F2RL3*_cg03636183 in buccal/saliva/bronchial washing samples warrants to be explored in further studies.

A major strength of the present study is its longitudinal design in which smoking-associated methylation markers were assessed in blood samples collected years before cancer diagnosis by pyrosequencing which is considered as the gold standard assay for DNA methylation at targeted sites. Furthermore, utmost care was given to correct for overoptimism by a split sample approach and cross-validation. In addition, detailed information on a variety of covariates was available and carefully controlled for in the analyses. A further strength is the follow-up of the study participants with regard to incident LC using data from the Saarland Cancer Registry which ensures an almost complete ascertainment of cancer cases in the population from which the study participants originated. Limitations of the study include the relatively small number of LC cases, in particular in stratified analyses, which restricted the study’s power. For example, the AUC for adenocarcinoma (0.830) was larger than the AUC for SCLC (0.739), but this difference did not meet the criterion for statistical significance. Future studies with sufficient numbers of histological subtypes of LC cases should address differences according to histological subtypes in more detail. Furthermore, only blood samples but no sputum or buccal samples were available in the ESTHER cohort. The performance of smoking-associated methylation markers from biospecimen directly exposed to tobacco smoke could therefore not be evaluated but deserves further investigation. Moreover, DNA methylation was quantified in whole blood samples without possibility for correction for leukocyte composition. However, the 3 target loci also exhibited the strongest associations with smoking in buccal cell DNA [19]. Blood cell composition therefore is unlikely to be a relevant issue in the current study.

## Conclusions

Despite its limitations, our study demonstrates that *AHRR*, *6p21.33*, and *F2RL3* methylation individually are strong predictors for lung cancer development. These markers therefore hold potentials to improve lung cancer diagnosis/screening either through incorporating them into promising screening panels or through risk stratification for further specific screening, such as CT examination.

## Additional file

Additional file 1: Table S1. The primers for the pyrosequencing analyses. **Table S2.** Associations of methylation at *AHRR*, *6p21.33*, and *F2RL3* with lung cancer risk in training set. **Table S3.** Smoking-history stratified associations of methylation at *AHRR*, *6p21.33*, and *F2RL3* with lung cancer risk. **Table S4.** Individual and joint performance of methylation at *AHRR*, *6p21.33*, and *F2RL3* in training and validation. **Table S5.** Optimism-corrected AUC (95% CI) among heavy and light smokers. **Figure S1.** Correlation between *AHRR*, *6p21.33*, and *F2RL3* methylation. (DOCX 222 kb)

## Abbreviations

AHRR: Aryl-hydrocarbon receptor repressor
AUC: Areas under the curve
BMI: Body mass index
CI: Confidence interval
CpG: Cytosine-phosphate-guanine
CT: Computed tomography
ESTHER: Epidemiologische Studie zu Chancen der Verhütung, Früherkennung und optimierten Therapie chronischer Erkrankungen in der älteren Bevölkerung
EWAS: Epigenome-wide association study
F2RL3: Coagulation factor II (thrombin) receptor-like 3
ICD-10: International Classification of Diseases-10
LC: Lung cancer
NSCLC: Non-small cell lung cancer
OR: Odds ratio
Ref.: Reference
ROC curves: Receiver operating characteristic curves
RSC regression: Restricted cubic spline regression
SCLC: Small cell lung cancer
SD: Standard deviation
