# Supplementary material for: Smoking-associated DNA methylation markers predict lung cancer incidence
Source: Clin Epigenetics. 2016 Nov 25;8:127. doi: 10.1186/s13148-016-0292-4 (PMC5123284; doi:10.1186/s13148-016-0292-4)
Supplement: Additional file 1: Table S1. — The primers for the pyrosequencing analyses. Table S2. Associations of methylation at AHRR, 6p21.33, and F2RL3 with lung cancer risk in training set. Table S3. Smoking-history stratified associations of methylation at AHRR, 6p21.33, and F2RL3 with lung cancer risk. Table S4. Individual and joint performance of methylation at AHRR, 6p21.33, and F2RL3 in training and validation. Table S5. Optimism-corrected AUC (95% CI) among heavy and light smokers. Figure S1. Correlation between AHRR, 6p21.33, and F2RL3 methylation. (DOCX 222 kb) [file 13148_2016_292_MOESM1_ESM.docx]

Supplementary Appendix to

**Smoking-associated DNA methylation markers predict lung cancer incidence**

Yan Zhang^1^, Magdeldin Elgizouli^2^, Ben Schöttker^1^, Bernd Holleczek^3^, Alexandra Nieters^2^, Hermann Brenner^1,4,5^

^1^Division of Clinical Epidemiology and Aging Research, German Cancer Research Center (DKFZ), Heidelberg, Germany; ^2^Centre of Chronic Immunodeficiency (CCI), Module Epidemiology, University Medical Center Freiburg, Freiburg, Germany; ^3^Saarland Cancer Registry, Saarbrücken; ^4^Germany Division of Preventive Oncology, German Cancer Research Center (DKFZ) and National Center for Tumor Diseases (NCT), Im Neuenheimer Feld 460, D-69120 Heidelberg, Germany; ^5^German Cancer Consortium (DKTK), German Cancer Research Center (DKFZ), Heidelberg, Germany

**Table S1. The primers for the pyrosequencing analyses**

**Table S2. Associations of methylation at *AHRR, 6p21.33*, and *F2RL3* with lung cancer risk in training set**

**Table S3. Smoking-history stratified associations of methylation at *AHRR*, *6p21.33*, and *F2RL3* with lung cancer risk**

**Table S4. Individual and joint performance of methylation at *AHRR, 6p21.33*, and *F2RL3* in training and validation**

**Table S5. Optimism-corrected AUC (95% CI) among heavy and light smokers**

**Figure S1. Correlation between *AHRR, 6p21.33*, and *F2RL3* methylation**

Table S1. The primers for the pyrosequencing analyses

| Marker | Primer |
| --- | --- |
| *AHRR* | F AGGGGTTGTTTAGGTTATAGATT  R b-CTCCAAAACCCCAAAAACCAACCTATC  S GTTTTGGTTTTGTTTTGTATT |
| *6p21.33* | F TTGGAGAATTTGATGGAGATTGAAGTTAA  R b-AAACTACAAACAAACCAACCCCAACA  S TTTTTTTGAAATTTTATGATTTAGT |
| *F2RL3* | F GGTTTATTAGTAGTATGGTGGAGGG  R b-CCCAAACCAAATCTATACCAATAACAATAAC  S TAGTATGGTGGAGGG |

F – forward; R – reverse; S – sequencing; b - biotin

Table S2. Associations of methylation at *AHRR, 6p21.33*, and *F2RL3* with lung cancer risk in training set

|  | Methylation level ^a^ / Smoking status | Controls | Cases | OR (95% CI) | | |
| --- | --- | --- | --- | --- | --- | --- |
|  |  |  |  | Model 1^b^ | Model 2^c^ | Model 3^d^ |
| *AHRR*_cg05575921 | ≥ 85 (Quartile 4) | 59 | 3 | Ref. | Ref. | Ref. |
|  | < 85 (Quartile 3) | 52 | 3 |  |  |  |
|  | < 80 (Quartile 2) | 56 | 14 | 6.36 (2.19 - 18.48) | 2.51 (0.69 - 9.11) | 2.35 (0.51 - 10.86) |
|  | < 68 (Quartile 1) | 55 | 56 | 26.93 (10.02 - 72.36) | 7.31 (1.97 - 27.17) | 6.44 (1.48 - 28.10) |
|  |  |  |  |  |  |  |
|  | Per SD less methylation |  | --- | 2.54 (1.95 - 3.32) | 1.49 (0.99 - 2.26) | 1.71 (1.04 - 2.80) |
|  |  |  |  |  |  |  |
|  | Never smoker | 86 | 5 | Ref. | Ref. | Ref. |
|  | Former smoker | 90 | 29 | 4.86 (1.46 - 16.16) | 2.42 (0.61 - 9.56) | 1.34 (0.29 - 6.27) |
|  | Current smoker | 40 | 43 | 11.02 (2.81 - 43.15) | 3.02 (0.61 - 14.99) | 2.19 (0.36 - 13.44) |
|  | Per 24 (= 1SD) pack-years |  | --- | 1.97 (1.23 - 3.14) | 1.72 (1.07 - 2.76) | 2.01 (1.13 - 3.59) |
| *6p21.33*_cg06126421 | ≥ 73 (Quartile 4) | 58 | 3 | Ref. | Ref. | Ref. |
|  | < 73 (Quartile 3) | 57 | 16 |  |  |  |
|  | < 66 (Quartile 2) | 53 | 27 | 3.31 (1.67 - 6.57) | 1.89 (0.82 - 4.37) | 1.92 (0.74 - 4.97) |
|  | < 57 (Quartile 1) | 54 | 30 | 3.59 (1.83 - 7.06) | 0.76 (0.31 - 1.90) | 0.88 (0.31 - 2.53) |
|  |  |  |  |  |  |  |
|  | Per SD less methylation |  | --- | 1.75 (1.33 - 2.31) | 0.93 (0.63 - 1.38) | 0.92 (0.59 - 1.43) |
|  |  |  |  |  |  |  |
|  | Never smoker | 86 | 5 | Ref. | Ref. | Ref. |
|  | Former smoker | 90 | 29 | 4.86 (1.46 - 16.16) | 4.63 (1.37 - 15.63) | 2.39 (0.63 - 9.13) |
|  | Current smoker | 40 | 43 | 11.02 (2.81 - 43.15) | 14.11 (3.34 - 59.54) | 8.36 (1.63 - 42.98) |
|  | Per 24 (= 1SD) pack-years |  | --- | 1.97 (1.23 - 3.14) | 1.97 (1.21 - 3.22) | 2.27 (1.26 - 4.10) |
| *F2RL3*_cg03636183 | ≥ 81 (Quartile 4) | 74 | 3 | Ref. | Ref. | Ref. |
|  | < 81 (Quartile 3) | 49 | 7 |  |  |  |
|  | < 78 (Quartile 2) | 47 | 11 | 3.22 (1.26 - 8.22) | 1.29 (0.44 - 3.8) | 1.20 (0.33 - 4.38) |
|  | < 73 (Quartile 1) | 52 | 55 | 14.88 (6.84 - 32.37) | 4.43 (1.64 - 11.97) | 6.56 (1.94 - 22.2) |
|  |  |  |  |  |  |  |
|  | Per SD less methylation |  | --- | 2.28 (1.76 - 2.94) | 1.44 (0.99 - 2.09) | 1.58 (1.01 - 2.46) |
|  |  |  |  |  |  |  |
|  | Never smoker | 86 | 5 | Ref. | Ref. | Ref. |
|  | Former smoker | 90 | 29 | 4.86 (1.46 – 16.16) | 2.82 (0.77 - 10.3) | 1.34 (0.31 - 5.92) |
|  | Current smoker | 40 | 43 | 11.02 (2.81 – 43.15) | 3.23 (0.66 - 15.79) | 1.65 (0.26 - 10.61) |
|  | Per 24 (= 1SD) pack-years |  | --- | 1.97 (1.23 – 3.14) | 2.05 (1.25 – 3.36) | 2.44 (1.32 – 4.50) |

^a^ Quartiles of each sites among controls in the training set; ^b^Model1: adjusted for age and sex; ^c^Model 2: like model 1, additionally adjusted for smoking status + pack-years/methylation quartiles; ^d^Model 3: like model 2, additionally adjusted for educational level, BMI, physical activity, systolic blood pressure, total cholesterol, family history of cancer, prevalence of hypertension, cardiovascular disease, and diabetes.

Abbreviations: OR, odds ratio; CI, confidence interval; Ref., reference category; SD, standard deviation.

Table S3. Smoking-status stratified associations of methylation at *AHRR, 6p21.33*, and *F2RL3* with lung cancer risk

| CpG site | Smoking status^a^ | OR (95% CI)^b^ | | |
| --- | --- | --- | --- | --- |
|  |  | Model 1^c^ | Model 2^d^ | Model 3^e^ |
| *AHRR*_cg05575921 | Light smoker | 1.82 (1.39 – 2.38) | 1.52 (1.05 – 2.21) | 1.41 (0.92 – 2.14) |
|  | Heavy smoker | 1.40 (0.97 – 2.01) | 1.23 (0.80 – 1.90) | 1.29 (0.81 – 2.07) |
|  |  |  |  |  |
| *6p21.33*_cg06126421 | Light smoker | 1.63 (1.19 – 2.22) | 1.34 (0.91 – 1.97) | 1.41 (0.92 – 2.16) |
|  | Heavy smoker | 1.04 (0.75 – 1.45) | 0.97 (0.68 – 1.38) | 1.07 (0.70 – 1.64) |
|  |  |  |  |  |
| *F2RL3*_cg03636183 | Light smoker | 2.03 (1.53 – 2.69) | 1.82 (1.25 – 2.65) | 1.65 (1.07 – 2.53) |
|  | Heavy smoker | 1.23 (0.86 – 1.78) | 1.07 (0.72 – 1.60) | 1.15 (0.71 – 1.87) |

^a^ Light smokers include participants with <30 pack-years of smoking or who quit smoking >15 years ago for former smokers; Heavy smokers include participants with ≥30 pack-years of smoking who were current smokers or quit smoking ≤15 years ago; ^b^OR for per standard deviation (SD) less methylation level of each CpG sites; ^c^Model1: adjusted for age and sex; ^d^Model 2: like model 1, additionally adjusted pack-years and time since quitting; ^e^Model 3: like model 2, additionally adjusted for educational level, BMI, physical activity, systolic blood pressure, total cholesterol, family history of cancer, prevalence of hypertension, cardiovascular disease, and diabetes.

Abbreviations: OR, odds ratio; CI, confidence interval.

Table S4. Individual and joint performance of methylation at *AHRR, 6p21.33*, and *F2RL3* in training and validation

| Markers | AUC (95% CI) | |
| --- | --- | --- |
|  | Training set | Validation set |
| *AHRR*_cg05575921 | 0.792 (0.736 – 0.848) | 0.799 (0.733 – 0.866) |
| *6p21.33*_cg06126421 | 0.662 (0.597 – 0.726) | 0.789 (0.725 – 0.853) |
| *F2RL3*_cg03636183 | 0.791 (0.735 – 0.846) | 0.812 (0.725 – 0.871) |
|  |  |  |
| β1×M*_AHRR_*+β2×M*_6p21.33_* +β3×M *_F2RL3_*+β4×M*_6p21.33_*×M *_F2RL3_^a^* | 0.829 (0.778 – 0.881) | 0.799 (0.737 – 0.861) |
|  |  |  |
| Smoking index^b^ | 0.797 (0.744 – 0.850) | 0.819 (0.759 – 0.880) |
|  |  |  |
| Methylation score^c^ | 0.771 (0.713 – 0.830) | 0.811 (0.753 – 0.870) |
|  |  |  |
| *AHRR*_Binary +*6p21.33*_Binary +*F2RL3*_ Binary^d^ | 0.818 (0.769 – 0.866) | 0.788 (0.725 – 0.850) |

^a^ βs derived from regression coefficients in the training set: β1=-.0685, β =.4673, β3 =.3173, and β4=-.00612;

^b^ Smoking index bases on 3 CpGs, computed according to algorithm introduced by Teschendorff et al. *JAMA Oncol.* 2015;1(4):476-485;

^c^ Methylation score constructed according to quartiles of controls in the training set, with value of 3, 2, 1, and 0, respectively, for participants in the lowest quartiles of all 3 CpGs, of 2 of the 3 CpGs, of 1 of the 3 CpGs, and others;

^d^ optimal cut points of each CpG determined by Youden's J Index, *AHRR*_Binary=1/ 0 if cg05575921 >74%/ ≤74%, *6p21.33***_**Binary=1/ 0 if cg06126421 >70%/ ≤70%, and *F2RL3*_ Binary =1/0 if cg03636183 >70%/ ≤70.

Abbreviations: AUC, areas under the curve; CI, confidence interval.

Table S5. Optimism-corrected AUC (95% CI) among heavy and light smokers

| Group | AUC (95% CI) | | | | |
| --- | --- | --- | --- | --- | --- |
|  | Pack-years (PY) | AHRR_cg05575921 | 6p21.33_cg06126421 | F2RL3_cg03636183 | combination^a^ |
| **Heavy smokers**  N=150 (77 lung cancer cases)  (97 current smokers with ≥30 PY +  53 former smokers with ≥30PY and quit≤ 15 years ago) | 0.563  (0.471 - 0.656) | 0.540  (0.445 - 0.635) | 0.504  (0.410 - 0.598) | 0.528  (0.433 - 0.622) | 0.587  (0.495 - 0.679) |
| **Light smokers**  N=231 (51 lung cancer cases)  (51 current smokers with <30 PY +  153 former smokers with <30 PY +  27 former smokers with ≥30 PY and quit >15years ago) | 0.561  (0.454 - 0.668) | 0.704  (0.618 - 0.789)  (p=0.04, vs. PY) | 0.617  (0.529 - 0.706) (p=0.42, vs. PY,) | 0.743  (0.666 - 0.820)  (p=0.007, vs. PY) | 0.747  (0.669 - 0.825) (p=0.006, vs. PY) |
| **Light smokers (Sensitivity analysis)^b^**  N=204 (41 Lung cancer cases)  (51 current smokers with <30 PY +  153 former smokers with <30 PY) | 0.491  (0.372 -0.610) | 0.700  (0.604 - 0.795) | 0.632  (0.537 - 0.729) | 0.755  (0.674 - 0.837) | 0.757  (0.673 - 0.840) |

^a^Combination formula: β1×M*_AHRR_*+β2×M*_6p21.33_* +β3×M*_F2RL3_*+β4× M*_6p21.33_* × β3×M*_F2RL3_*=(-0.0685) ×cg05575921 + 0.4673 × cg06126421 + 0.3173 × cg03636183 + (-0.00612) × cg06126421 × cg03636183, where underlined coefficients were derived from regression coefficients in training set. ^b^A sensitivity analysis was conducted among light smokers after excluding participants with ≥30 pack-years and quit smoking >15years ago.

Abbreviations: AUC, areas under the curve; CI, confidence interval; PY, pack-years.

Figure S1. Correlation between *AHRR, 6p21.33*, and *F2RL3* methylation

Training set Validation set


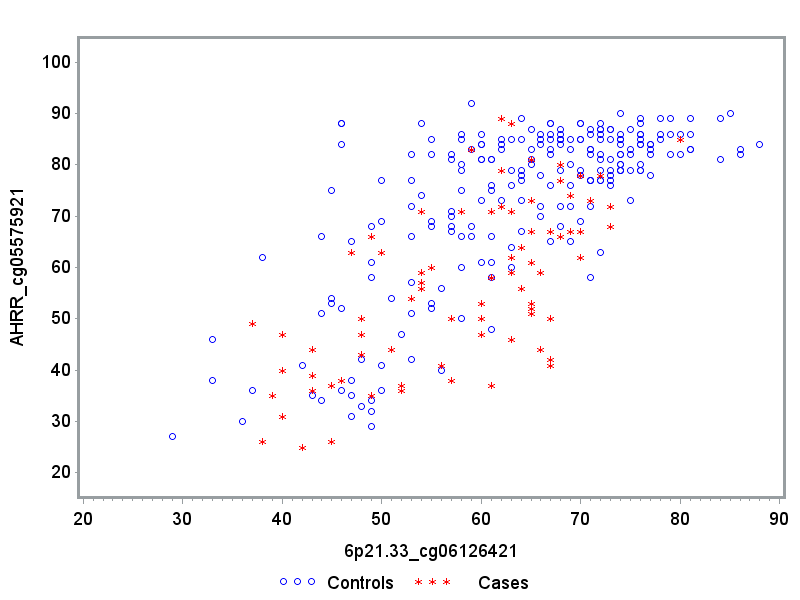

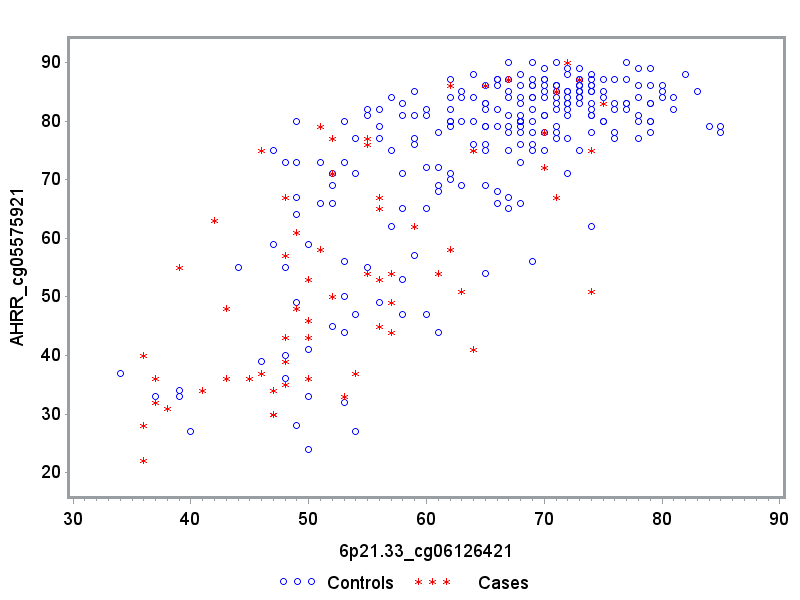


r = 0.6587

r = 0.6594

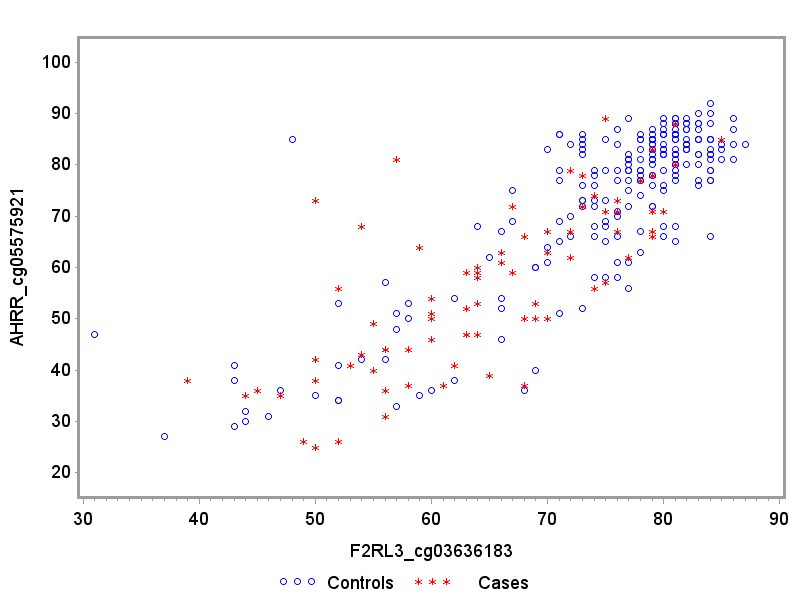

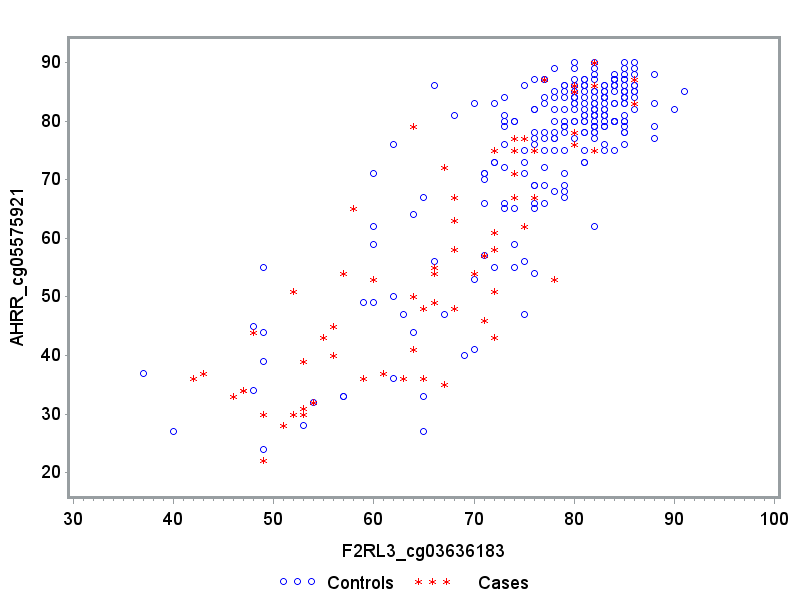


r = 0.7244

r = 0.7867


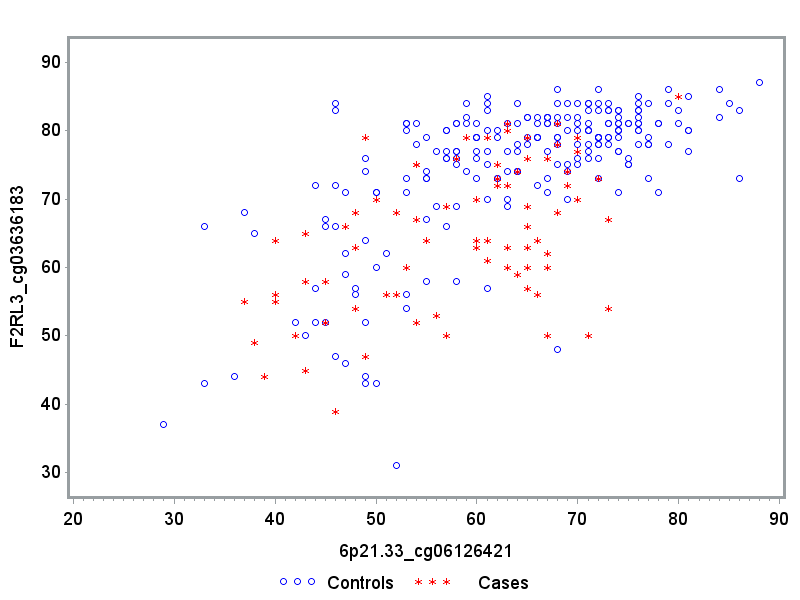

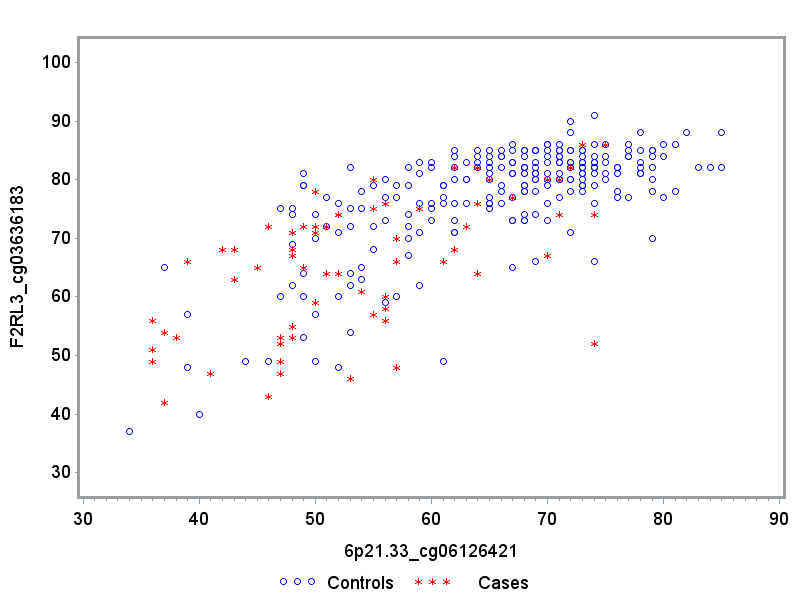


r = 0.6293

r = 0.6178
